# Recipient natural killer cells alter the course of rejection of allogeneic heart grafts in rats

**DOI:** 10.1371/journal.pone.0220546

**Published:** 2019-08-22

**Authors:** Oliver Beetz, Joline Kolb, Benjamin Buck, Britta Trautewig, Kai Timrott, Florian W. R. Vondran, Ingrid Meder, Corinna Löbbert, Joachim Hundrieser, Jürgen Klempnauer, Hüseyin Bektaş, Thorsten Lieke

**Affiliations:** 1 Regenerative Medicine and Experimental Surgery, Department of General-, Visceral- and Transplantation Surgery, Hannover Medical School, Hannover, Germany; 2 Transplant Laboratory, Department of General-, Visceral- and Transplantation Surgery, Hannover Medical School, Hannover, Germany; 3 Department of General-, Visceral- and Oncological Surgery, Hospital Group Gesundheit Nord, Bremen, Germany; Centro Cardiologico Monzino, ITALY

## Abstract

Rejection of solid organ grafts is regarded to be dependent on T cell responses. Nonetheless, numerous studies have focused on the contribution of NK cells in this process, resulting in contradictory theories. While some conclude that there is no participation of NK cells, others found an inflammatory or regulative role of NK cells. However, the experimental settings are rarely comparable with regard to challenged species, strain combinations or the nature of the graft. Thus, clear definition of NK cell contribution might be impeded by these circumstances. In this study we performed heterotopic heart transplantation (HTx) in rats, choosing one donor-recipient-combination leading to a fast and a second leading to a prolonged course of graft rejection. We intervened in the rejection process, by depletion of recipient NK cells on the one hand and by injection of activated NK cells syngeneic to the recipients on the other. The fast course of rejection could not be influenced by any of the NK cell manipulative treatments. However, the more prolonged course of rejection was highly susceptible to depletion of NK cells, resulting in significant acceleration of rejection, while injection of NK cells induced acceptance of the grafts. We suggest that, depending on the specific setting, NK cells can attenuate the first trigger of immune response, which allows establishing the regulatory activity leading to tolerance of the graft.

## Introduction

The participation of NK cells in rejection of solid organ grafts was first described in the early 1980s, with evidence of influx of NK cells, then also described as large granular lymphocytes (LGL) into rat renal allografts [1]. Shortly thereafter the missing self-theory postulated by Kaerre et al. gave a reasonable explanation, why NK cells should respond to foreign grafts: Cells that lack expression of self MHC-I molecules are targets for NK cell mediated killing [2].

However, a study by Shelton et al. reported of non-rejection of allogeneic skin-grafts in SCID mice, whereas rejection was re-established by transfer of–predominantly CD4+–T cells [3], prompting the conclusion that the adaptive immune system plays the major role in the rejection process towards foreign tissue.

Meanwhile, it is a common consent that an effective and in best case specific prevention of graft rejection needs to abrogate the onset of an allogen-reactive T cell response. NK cells are often considered as a rather negligible if not redundant cell population in this process [4,5].

On the other hand, there is a group of reports providing evidence that NK cells do fulfil a role in immune responses against allogeneic grafts [6,7]. A prominent and often cited report from Maier et al., using CD28^-/-^ mice, in which a T cell activation is suspended, but rejection of a solid organ graft can still be observed–as long as recipient NK cells are present [8]. Furthermore, recipient’s NK cells were shown to kill donor-derived passenger antigen-presenting cells (APC) [9] and allo-specific responses by NK cells can be detected [10,11]. Additionally, the invasion of graft tissue by NK cells, which was first described approximately 35 years ago with the vague term of infiltrating LGL cells, has also been reported more recently in a study of liver transplantation in rats [12] and in mice after skin transplantation [13].

These data provide evidence that NK cells recognize and respond against allogeneic grafts according to the missing-self theory. However, under conditions of established T cell activity NK cell responses seem to be of less importance and influence.

A comparatively new aspect of NK cell immunology is their ability to not only react in an inflammatory or cytotoxic manner, but also to regulate and suppress immune responses. NK cells secrete interleukin (IL)-10 [14,15] and tumour growth factor (TGF)-β [11,16] with the ability to reduce proliferation of CD4^+^ T cells in a direct interaction [16]. In some diseases this influence of NK cells attenuates the T cell-mediated inflammatory damage and leads to favourable host outcome [17]. However, an effective suppression of the adaptive immune response is only successful before the activation process of T cells has been initiated, as was also described by our workgroup [16].

The aim of the present study was to evaluate the hypothesis that NK cells react to allogeneic grafts and upon activation interfere with T cells in a suppressive manner. For this purpose, we depleted recipient NK cells before heterotopic heart transplantation (HTx) in rats or injected exogenous, syngeneic NK cells directly after successful HTx.

## Materials and methods

### Ethics

All animal experiments were performed according to protocols approved by the local Ethics Animal Review Board of the regional authorities for consumer protection and food safety of Lower Saxony (LAVES, Oldenburg, Germany) with the approval ID 12/0768.

### Animals

Lewis wild-type (Lew wt, RT1^l^) rats were purchased from Charles River Laboratories (Sulzfeld, Germany). Lewis.1a (RT1^a^) and Lew.1u7B (RT1^u^) were bred in the animal facility of the Hannover Medical School. The Lew.1u7B strain (formally designated as Lew.1W-7B) is MHC identical with Lew.1u rats, but expresses the congenic marker CD45.2 (RT7^b^) which causes no differences in immunological characteristics compared to Lew.1u rats [18]. All rats were 10–12 weeks old and the deployed strains were completely MHC (class I and II)-disparate among each other.

### Heterotopic heart transplantation

The method performed is based on the work of Lindsey and Ono [19]. The rats were anaesthetised using a mobile isoflurane vaporising unit (Summit Anesthesia Solutions, Bend, Oregon). The donor was intravenously injected with 500 I.U. heparin, the thorax was subsequently opened and the ascending aorta and the pulmonary trunk were severed. The inferior and superior vena cava, as well as the pulmonary veins, were ligated with a single 5–0 ligature. The explanted heart was perfused with 30 ml isotonic saline solution supplemented with 1000 I.U. heparin. For implantation the abdominal aorta and the inferior vena cava were temporarily clamped using a Cooley vascular clamp and the vessels were incised in longitudinal direction. The donor ascending aorta was connected to the abdominal aorta and the donor pulmonary trunk to the inferior vena cava via 8–0 monofilament suture. For perioperative analgesia, rats were treated with a subcutaneous depot of 5 mg Carprofen per kilogram bodyweight. Graft survival was controlled by daily palpation through the abdominal wall.

### Treatment of rats prior and after transplantation

#### *In vivo* depletion of NK cells using mAb HT30

Monoclonal antibody (mAb) HT30 detects the allomorphic protein NKR-P1A^LEW^. It has been developed in our lab, using the rat strain pair LEW.TO-NKC2 (donor, carries *NKR-P1A*^*LEW*^) and LEW.TO-NKC (recipient, carries *NKR-P1A*^*TO*^). Both NKC-congenic strains share the same genetic background and allelic sequences of the natural killer gene complex (NKC) downstream of *NKR-P1A*. LEW.TO-NKC animals were immunized with donor spleen cells. Supernatants of established hybridoma cells were screened using 293 cells transfected with NKR-P1A^LEW^.

NK cells were depleted *in vivo* by a singular subcutaneous application of 500 μg of mAb HT30 one day prior to HTx.

#### Injection of NK cells

NK cells were positively isolated using biotinylated mAb 3.2.3 and Streptavidin-labelled microbeads (Miltenyi Biotec, Bergisch Gladbach, Germany). The isolated NK cells were incubated for 7–10 days in culture medium supplemented with rat IL-2. 1-2x10^6^ NK cells (with a purity ≥ 90%) were then injected intravenously as a single shot directly after transplantation.

#### Treatment with Ciclosporin (CsA)

Selected recipients were injected daily with a subtherapeutic dose of 1.25 mg/kg body-weight subcutaneously. This treatment led to 60% graft survival after the first 21 days of observation.

### Subcutaneous placement of heart cells in the ear

Perfused explanted hearts from donors were chopped up into 3x3 mm blocks and digested with 0,5 mg/ml collagenase I for 30 min at 37°C. The tissue was mashed through a large-pore sieve, resulting in vital muscle cell congeries, (mostly dead) single heart cells and remaining blood cells. By passage through a 40 μm cell strainer, the congeries were separated and 1x10^4^ were injected subcutaneously in the ear of specified recipients.

### Histology and scores for infiltration

Cryostat sections of Tissue-Tek (Sakura, Alphen aan den Rijn, Netherlands) embedded grafts were air dried, acetone/methanol fixed, and incubated with mAb to TCRα/β (clone R73), CD4 (W3/25), CD68 (ED1, AbD Serotec, Düsseldorf, Germany), CD161 (3.2.3) and NKR-P1A (HT30). Antibodies were purified in our lab, except where noted. Stained cells were detected with bridge antibodies (rabbit anti-mouse Ig) and alkaline phosphatase anti-alkaline phosphatase (APaAP) (both Dako, Hamburg, Germany). Nuclear staining of sections was performed with hematoxylin (Merck, Darmstadt, Germany). Infiltration of lymphocytes was assessed in double-blind evaluation by light. Of note, our quantification followed a fixed classification starting with 0.5 = singular distributed positive stained cells marginally occurring in the tissue section; 1.0 = singular distributed positive stained cells occurring in every field of view; 1.5 = numerous positive stained cells uniformly distributed over the whole tissue section; 2.0 = strong distribution and 2.5 = very strong distribution of positive stained cells.

### Flow cytometry

Cell populations were stained with mAb against CD4 (W3/25), CD8 (Ox8), CD161 (10/78), TCR α/β chain (R73), CD25 (Ox39), CD86 (24F), CD11b/c (Ox-42) and CD172a (Ox41) (BioLegend, London, UK).

### Mixed lymphocyte reaction

2x10^5^ responder cells were either stimulated and re-stimulated applying a specific stimulus with equal numbers of lethally irradiated allogeneic splenocytes or using plate-bound CD3 and soluble CD28. After 5 days incubation in 96 well-round bottom plates, lymphocytes were pulsed with 0.5–1 μCi [^3^H]thymidine/well for 16 hours and [^3^H]thymidine incorporation was assessed after scintillation using a β-counter (LKB Wallac, Turku, Finland). In certain mixed cultures, NK cells were depleted from bulk splenocytes using mAb 3.2.3 and MACS beads. IFN-γ production was measured in the supernatants of the culture by enzyme-linked immunoabsorbant assay (ELISA).

### Assessment of mRNA expression

Draining cervical lymph node (LN) cells from Lew wt and Lew.1a rats grafted with allogeneic heart cells in the ear were lysed and mRNA was isolated using NucleoSpin RNA II kit (Macherey-Nagel, Düren, Germany) according to manufacturer’s instructions. mRNA was subsequently translated into complementary DNA by supplementing 1 μg RNA with Oligo(dT) primer and RevertAid Transcriptase (both Fermentas, St. Leon-Rot, Germany) and incubation for 1 h at 42°C. Reaction was stopped at 72°C for 10 min. cDNA was amplified by PCR using primer for TGF-β, IL-10, FoxP3, IL-1β and IFN-γ (Table 1). Expression of GAPDH served as housekeeping gene. PCR included 30 cycles with 30 sec 94°C, 30 sec 58°C and 1 min 30 sec 72°C.

**Table 1:** Molecules and primer sequences for PCR analysis.

molecule	forward	reverse	Reference
GAPDH (200bp)	CCTTCATTGACCTCAACTACATG	CTTCTCCATGGTGGTGAAGAC	[20]
TGF-β (430bp)	CCGCAACAACGCAATCTA	TGAGGAGCAGGA AGGGTC	[20]
IL-10 (160bp)	CCTGCTCTTACTGGCTGGAG	TCCAGCTGGTCCTTCTTTTG	[21]
FoxP3 (570bp)	GCACAAGTGCTTTGTGCGAGT	TGTCTGTGGTTGCAGACGTTGT	[22]
IL-1β (80bp)	GACCTCTCAAGCAGAGCACAG	GGGTTCCATGGTGAAGTCAAC	[23]
IFN-γ (220bp)	GCCCTCTCTGGCTGTTACTG	CTGATGGCCTGGTTGTCTTT	[24]

Analyzed molecules and specific primer sequences for PCR analysis and respective references

### Statistical analysis

Results are presented in general as the mean ± standard deviation, depicting at least five individuals per group. *In vitro* experiments were performed independently usually five to six times (minimum of three times). Statistical analysis was generally performed with the unpaired two-tailed Student’s *t*-test using prism software (Graph Pad Software, San Diego, CA, USA).

Significance is indicated with * for p-values ≤ 0.05, ** for p-values ≤ 0.005 and *** for p-values ≤ 0.0005. If no asterisk is shown in the respective graph, the results were not significantly different.

## Results

### The comparison of two different, fully MHC disparate rat strain combinations discloses divergent effects on NK cells

To assess the possible regulative function of NK cells in allograft rejection *in vivo*, we performed heterotopic HTx for two reasons: 1. Graft survival can be assessed directly and non-invasively until final cessation of heartbeat, since graft rejection does not impede vital functions of the recipient. 2. We were able to refer to a comprehensive study by Klempnauer et al. who followed heart-graft survival in numerous MHC-disparate rat strains that were combined in a vice-versa manner [25].

We included a fast rejecting donor-recipient combination and a combination of strains resulting in a prolonged rejection, to analyze the regulatory capacity of NK cells, depending on the severity of the immune response. Hence, we chose the strain combination Lew.1a → Lew wt with a reported mean graft rejection of 8.4 days and the strain combination Lew.1u7B → Lew.1a, with a mean rejection time of 17.5 days [25].

**Fig 1:**
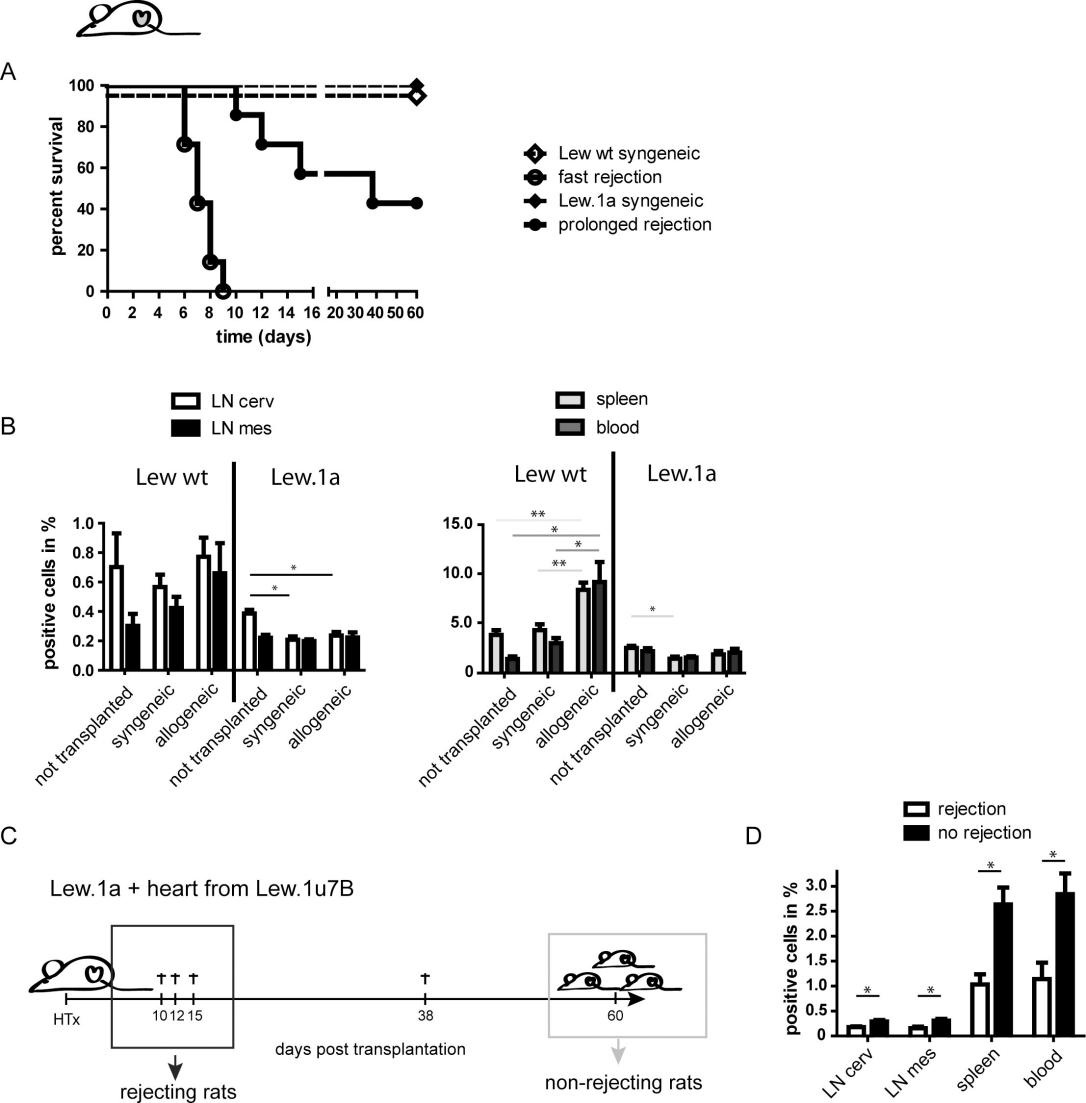
NK cells are affected differently by allogeneic grafts depending on the course of rejection. An illustration displaying a rat with a highlighted heart indicates that the presented data resulted from the model of HTx in distinction from data obtained from the second transplantation model, in which allogeneic heart muscle cells were injected into the ear of the respective recipients (see also Fig 3). (A) Perfused hearts derived from Lew.1a (RT1^a^) rats were heterotopically transplanted into the abdomen of Lew wt (RT1^l^) recipients (fast rejection) and Lew.1u7B (RT1^u^) hearts were engrafted in Lew.1a (RT1^a^) recipients (prolonged rejection). Syngeneic HTx served as a control group (n = 5). (B) Flow cytometric assessment of frequencies of NK cells in Lew wt and Lew.1a recipients after syngeneic and allogeneic HTx compared to naïve rats, respectively. The data implies divergent reactions of the two strains to the grafts: Upon allogeneic engraftment Lew wt recipients displayed increased NK cell ratios, which was observed especially in spleen and blood of the recipients, whereas Lew.1a recipients reacted with a general decrease of NK cell ratios compared to naïve rats or recipients of syngeneic grafts. (C) Three of 7 Lew.1a recipients receiving allogeneic grafts developed prolonged graft survival with no differences in viability, when compared to syngeneic controls over the complete observation period of 60 days resulting in the prolonged mean survival of allogeneic grafts in Lew.1a recipients. (D) NK cell frequencies of rejecting and non-rejecting Lew.1a recipients in cervical (cerv) and mesenterial (mes) LN, spleen and blood. Rejecting rats showed a significant decrease of NK cells, when compared to non-rejecting (and untreated) rats. Survival of grafts is shown as a Kaplan-Meier presentation (A) and in a diagram, respectively (C). The ratios of NK cells are presented as mean ± SEM of three (in D) and at least five (in B) recipients. Significance is indicated with * for p-values ≤ 0.05, ** for p-values ≤ 0.005 and *** for p-values ≤ 0.0005. If no asterisk is shown in the respective graph the results were not significantly different.

Allogeneic grafts from Lew.1a donors were rejected by Lew wt recipients in a mean time of 7.3 days, in line with results obtained by Klempnauer et al. [25], whereas syngeneic grafts showed vital function over the complete observation period (Fig 1A).

In Lew.1a recipients of allogeneic grafts, derived from Lew.1u7B donors, we observed a mean graft survival of 36.4 days, which was significantly longer than reported in the aforementioned study (Fig 1A). Of note, three of the seven Lew.1a recipients had vital graft function over the observation period of 60 days (Fig 1A and 1C).

Analysis of NK cell populations derived from lymphatic tissue (lymph node and spleen) and the blood of both recipient strains revealed that fast rejecting Lew wt recipients reacted with an increase of NK cell ratios upon allogeneic engraftment, when compared to naïve rats, accentuated in the spleen and blood. Lew.1a recipients on the other hand, displayed a general decrease of NK cell ratios compared to naïve rats (Fig 1B). A detailed comparison of rejecting and non-rejecting Lew.1a recipients demonstrated significantly lower NK cell ratios in the analyzed compartments in rejecting recipients (Fig 1D, S1A Fig).

Assessment of recipient T cell ratios in the two strain combinations also revealed distinct alterations: Whereas Lew wt rats engrafted with allogeneic hearts responded with a significant decrease of T cells in spleen and blood compared to the naïve rats or recipients of syngeneic grafts, Lew.1a recipients showed only moderate changes in T cell ratios upon allogeneic engraftment (S1B Fig). The specific alterations of NK cell ratios between the recipients of the two strains (Lew wt and Lew.1a) and the distinct results derived from rejecting and non-rejecting recipients in the latter strain, prompted us to investigate general differences in NK cell biology between the specific rat strains.

### NK cells show varying potential to influence the response of T cells depending on the specific rat strain

**Fig 2:**
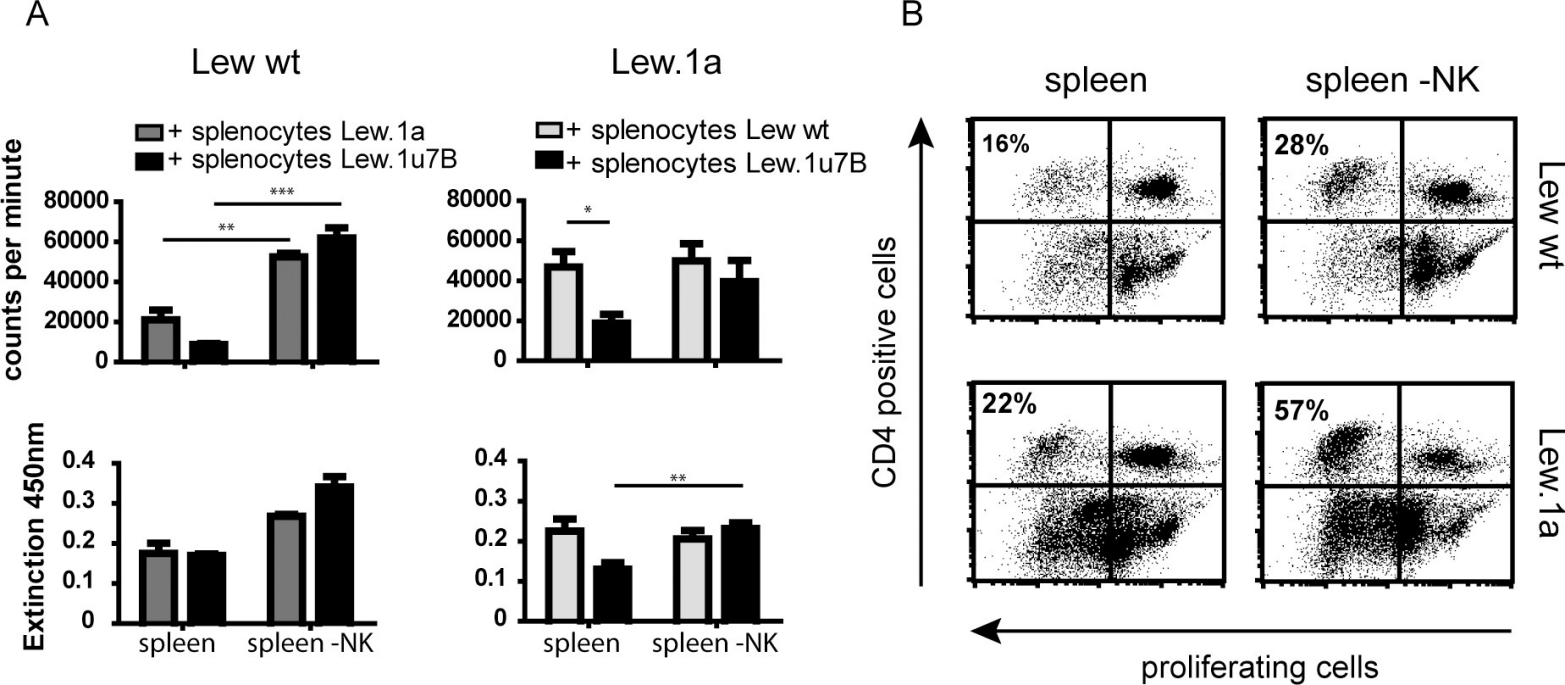
Distinct capacity of NK cells to control T cell activation, depending on the rat strain. (A) Proliferation (upper panel) and IFN-γ release (lower panel) of 2x10^5^ naïve splenocytes derived from Lew wt and Lew.1a rats after *in vitro* stimulation with 2x10^5^ irradiated splenocytes from the respective MHC disparate strains for 6 days. Responder splenocytes were either bulk or depleted of NK cells. The proliferation of T cells was highly controlled by NK cells in Lew wt rats, while Lew.1a rats showed a less consistent outcome in the absence of NK cells. The amount of secreted cytokine was highly related to the degree of proliferation, which indicates T cells as source of the detected IFN-γ. (B) Spontaneous proliferation of CMFDA-labelled Lew wt and Lew.1a splenocytes in the presence or absence of NK cells, respectively, incubated in medium alone for 6 days, was elevated in the absence of NK cells in both rat strains. Results are presented as exemplary dot blots or as mean ± SEM of at 4 least independent *in vitro* experiments. Significance is indicated with * for p-values ≤ 0.05, ** for p-values ≤ 0.005 and *** for p-values ≤ 0.0005. If no asterisk is shown in the respective graph the results were not significantly different.

Bulk splenocytes and those depleted of NK cells from Lew wt and Lew.1a rats were stimulated *in vitro* with irradiated splenocytes derived from the involved allogeneic rat strains of this study (cells derived from Lew.1u7B rats were only used for stimulation). The stimulation of untreated bulk splenocytes disclosed a highly distinct reaction to the respective trigger. Lew wt cells responded to Lew.1a splenocytes with higher proliferation (mean of 21 000 cpm) than to the Lew.1u7B strain (mean 8700 cpm). Lew.1a responder cells revealed a strong proliferation of about 47 000 cpm provoked by irradiated Lew wt cells, whereas the stimulation with Lew.1u7B derived stimulators resulted in a proliferation of 19 000 cpm (Fig 2A, upper panel). These strain dependent differences became more pronounced after depletion of NK cells from the responding splenocytes: Responder cells from Lew wt rats showed enhanced proliferation (> 2 times higher after stimulation with Lew.1a cells compared to bulk splenocytes; > 7 times higher in case of stimulation with Lew.1u7B cells). In responder cells of Lew.1a, the absence of NK cells resulted in almost no divergent proliferation after exposure to Lew wt stimulators (bulk: 46 800 cpm vs. NK depleted: 49 900 cpm), but in a > 2 times increase after stimulation with Lew.1u7B splenocytes, reflecting the strain combination used *in vivo*.

This potential influence on T cell proliferation by NK cells could also be observed by evaluation of IFN-γ release after NK cell depletion (Fig 2A, lower panel), which was increased in both responding strains.

Contrary to the allogeneic induction of proliferation, the assessment of spontaneous activation of T cells by incubation of splenocytes solely in medium induced a pronounced proliferation of CD4^+^ cells derived from Lew wt and especially from Lew.1a rats. This tendency was accentuated in the absence of NK cells (Fig 2B).

Analyzing the process of initiation of immune responses in Lew wt and Lew.1a recipients towards allogeneic grafts *in vivo* is challenging, especially since the explicit identification of graft draining LN is difficult in heterotopic HTx. As a consequence, the crucial local environment to follow the commencement of T cell activation after engraftment cannot be assessed.

Before manipulating NK cells in recipients of allogeneic heart grafts, we therefore performed additional *in vivo* experiments by challenging the two recipient strains by placing clusters of allogeneic heart muscle cells subcutaneously in the ear of the respective rat strain. By applying this method it is more feasible to track immunological processes in cervical lymph nodes (LN) draining the pinna.

### Subcutaneous placement of heart muscle cells induces an attenuated immune response in Lew.1a rats

**Fig 3:**
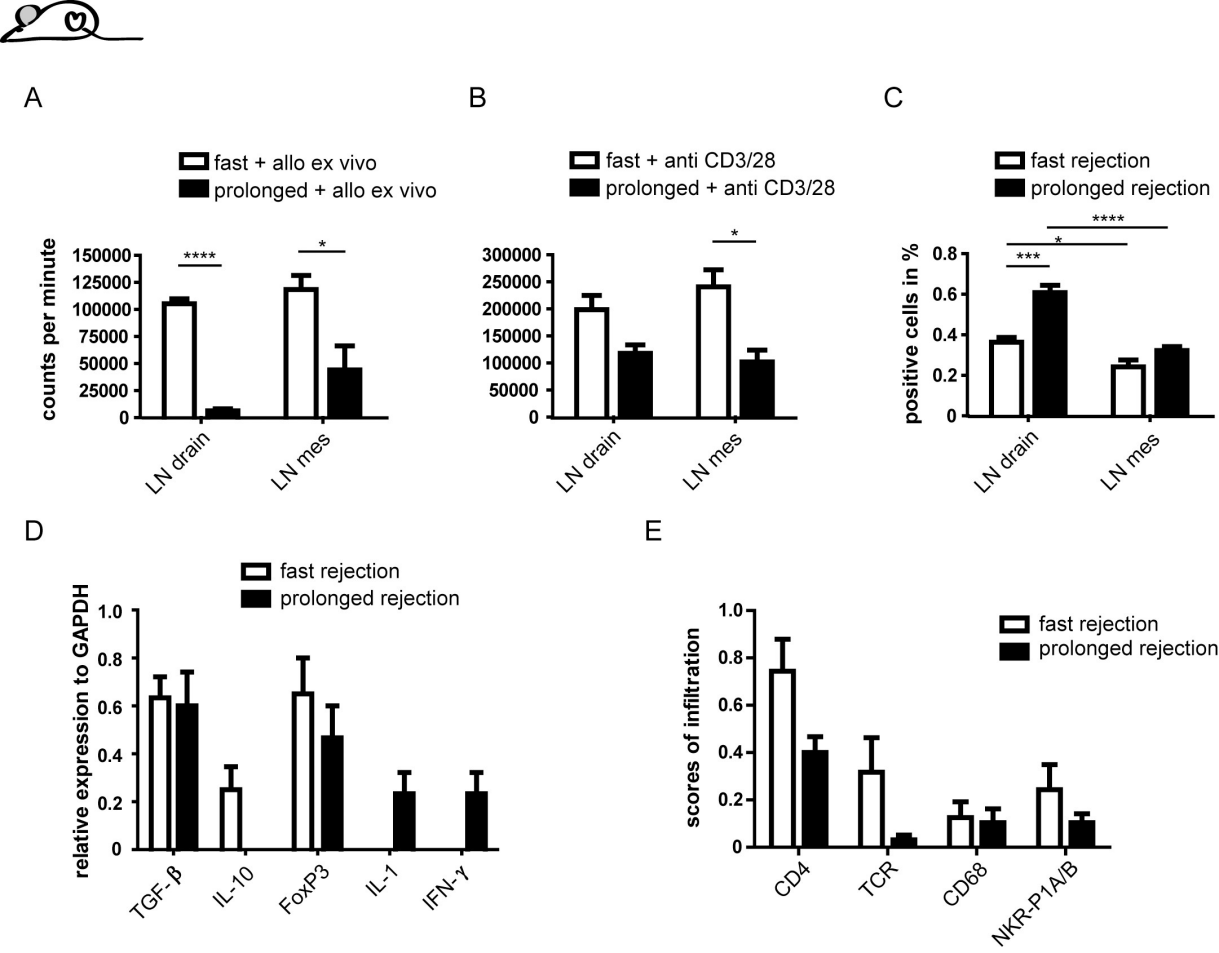
Lew.1a draining LN cells reveal increased NK cell ratios and suppressed proliferation upon specific re-stimulation. Lew wt and Lew.1a rats received allogeneic cell transplantation of 1x10^4^ small aggregates of muscle cells from collagenase digested heart tissue subcutaneously in the ear (Lew.1a heart cells → Lew wt (fast); Lew.1u7B heart cells → Lew.1a recipients (prolonged)). Eight days after Tx rats were sacrificed. Draining cervical LN and (as intrinsic control) mesenteric LN were prepared and tested in MLR for proliferative response and in flow cytometry for NK cell proportions, respectively. (A) Proliferation analyses of 2x10^5^ lymphocytes derived from draining cervical and mesenteric LN of Lew wt and Lew.1a rats after specific re-stimulation with 2x10^5^ irradiated splenocytes of the graft donor strain showed a significantly reduced proliferation of LN cells derived from Lew.1a rats when compared to Lew wt rats. (B) Unspecific stimulation of the lymphocytes with plate-bound mAb anti-CD3 and soluble anti-CD28 revealed general capacity of the lymphocytes to proliferate. (C) Percentages of NK cells in draining LN of Lew wt and Lew.1a recipients 8 days post placement of allogeneic heart cells in the ear. We observed a significant increase of NK cells in draining LN of Lew wt and significantly more pronounced in Lew.1a rats, when compared to mesenteric control LN. (D) mRNA expression in draining LN cells identical to the afore presented analyses in A-C. The cells were transferred into lysis buffer immediately after preparation and after mRNA purification subsequently transformed into cDNA. The outcome of a more suppressive cytokine-profile in Lew wt in comparison to a more inflammatory profile in Lew.1a recipients was surprising. (E) Scores of infiltrating CD4^+^, TCR^+^, CD68^+^ and NKR-P1A/B^+^ cells into ear tissue with regard to infiltration in syngeneic controls 8 days after Tx. The assessment revealed a clearly higher infiltration in Lew wt recipients in particular of T cells and monocytes. However, the infiltration of immune cells in general was very low. Results are presented as mean ± SEM of 4 individuals per group. Significance is indicated with * for p-values ≤ 0.05, ** for p-values ≤ 0.005, *** for p-values ≤ 0.0005 and **** for p-values ≤ 0.0001. If no asterisk is shown in the respective graph the results were not significantly different.

After subcutaneous placement of heart muscle cells in the pinna, cells harvested from (draining) cervical and mesenteric LN (serving as intrinsic control) were re-stimulated specifically with splenocytes from the respective donor strain. In Lew wt rats, re-stimulation led to an increase in proliferation, however, there was almost no detectable distinction in proliferation of lymphocytes derived from draining and mesenteric LN. In contrast, Lew.1a draining LN cells revealed strongly suppressed proliferation in comparison to mesenteric LN control cells and to draining LN from Lew wt (Fig 3A, S2 Fig). This suppression is mainly based on a significant reduction of activated T cells and not on T cell division in general (S2 Fig). Interestingly, unspecific activation of lymphocytes using CD3/28 antibodies induced pronounced proliferation in all tested LN of both strains, revealing a general proliferative capacity of T cells, even in Lew.1a draining (cervical) LN (Fig 3B, S2 Fig).

Parallel to these observations, relative NK cell numbers from draining LN of Lew.1a rats were significantly increased, when compared to intrinsic (mesenteric) control LN and draining LN from Lew wt (Fig 3C). Of note, the draining LN of Lew wt rats also revealed a significant increase of the NK cell ratio, when compared to the mesenteric LN, although this was much less accentuated than in Lew.1a recipients (p-value LN drain vs LN mes in Lew.1a: p = ≤ 0.0001 and Lew wt: p = 0.0145).

We took aliquots from the draining LN cells used for analysis of lymphocyte proliferation and NK cell ratios, prepared the cell mRNA and subsequently the corresponding cDNA. Cells derived from Lew wt rats had higher mRNA expression for IL-10 and FoxP3, but revealed a complete lack of IL-1 and IFN-γ expression (Fig 3D). In contrast, LN cells derived from Lew.1a rats displayed lower FoxP3 mRNA levels and no IL-10 but low levels of IL-1 and IFN-γ. Only the detected amounts of TGF-β were almost identical in both rat strains. Thus, regardless of the significantly higher proliferative activity of Lew wt T cells harvested from draining LN, a classical suppressive cytokine milieu was observed in the draining LN. Cells derived from Lew.1a rats displayed a more inflammatory profile according to the IL-1 and IFN-γ expression, although specific re-stimulation revealed reduced proliferative capacity.

The increased expression of suppressive cytokine mRNA could also indicate an enhanced immune response in Lew wt rats. For that reason, we also analyzed the infiltration of immune cells at the site of heart muscle cell placement in the pinna by immunohistochemistry, as an indication of the existence of activated immune cells. Indeed, Lew wt rats revealed a well detectable infiltration of immune cells, predominantly T cells and monocytes. In Lew.1a rats the influx of immune cells into the ear tissue was only marginal (Fig 3E). Thus, Lew.1a recipients challenged with Lew.1u7B cells displayed an attenuated immune response against the allogeneic stimulus, accompanied by an increase of NK cell ratios when compared to the response of Lew wt recipients to Lew.1a heart cells. The obtained cytokine profiles showed a simultaneous increase of IL-1 and IFN-γ, possibly accounting for an increase in activation of NK cells. However, the secretion of regulatory cytokines such as IL-10 and TGF-β in this specific setting could not be observed. Considering the initial *in vivo* as well as the above mentioned *in vitro* results, NK cells seem to exert specific immunoregulatory function in Lew.1a rats upon allogeneic challenge to Lew.1u7B cell or organ grafts.

### Successful interference on the course of allogeneic graft rejection by manipulation of NK cells in Lew.1a but not in Lew wt recipients

To further characterize NK cell functions *in vivo* we performed HTx in the above mentioned strain combinations and manipulated NK cells in the recipients either by depletion of recipient NK cells before HTx using our lab-made mAb HT30 (which is specific for NKR-P1A and does not cross-react with NKR-P1B) or by injection of activated syngeneic NK cells subsequently after successful surgery. The depletion of NK cells was highly efficient in both strains (S3 Fig). The injection of NK cells led to a decrease of NK cell ratios in Lew wt rats, whereas Lew.1a recipients did not reveal detectable alterations of NK cells ratios in the analyzed compartments (S3 Fig).

**Fig 4:**
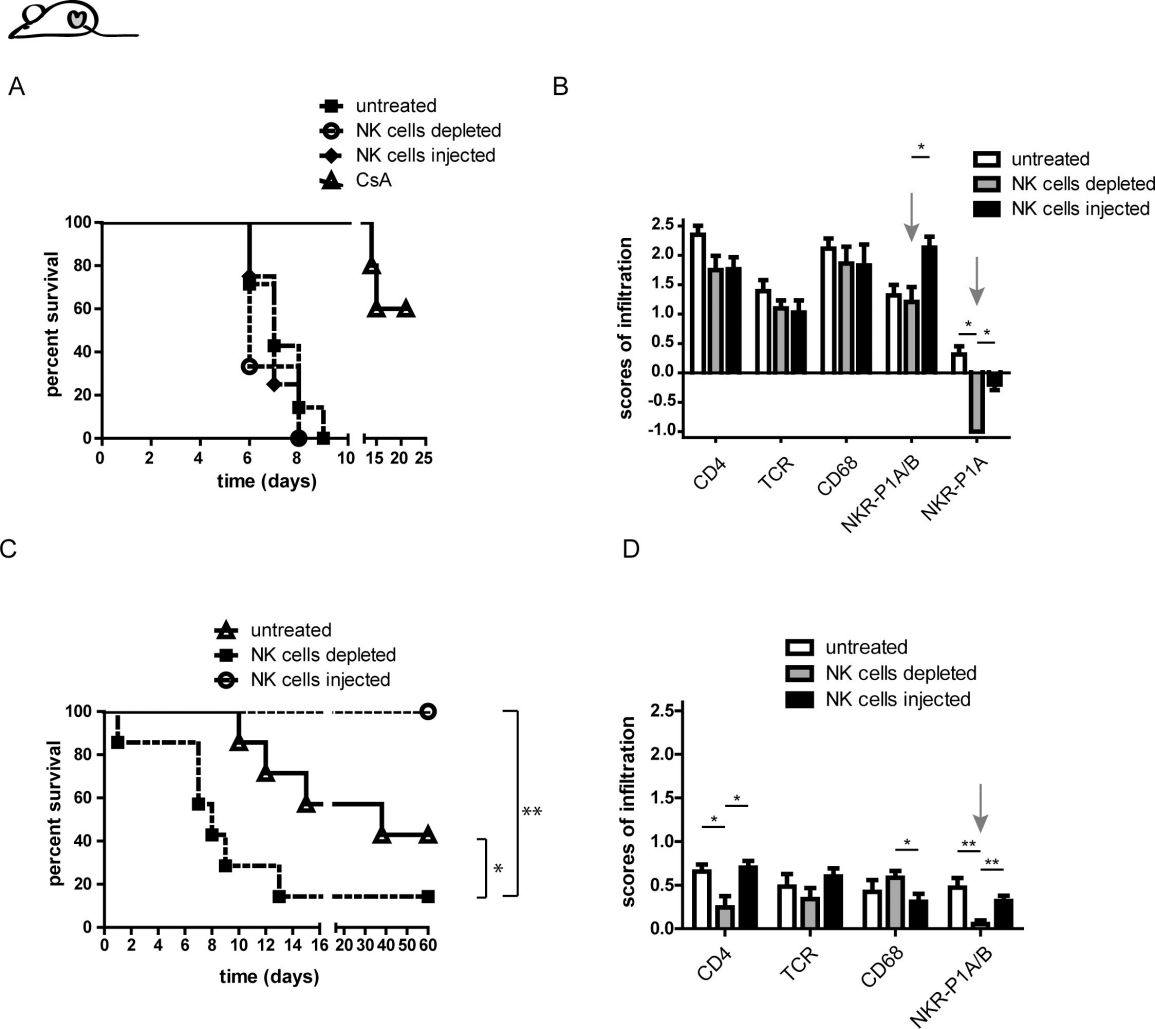
Heart graft rejection in Lew.1a recipients is significantly influenced by the presence of NK cells. (A) Kaplan-Meier presentation of allogeneic graft survival in Lew wt recipients without further treatment (untreated), with a single subcutaneous application of 500 μg HT30 one day prior HTx (NK cell depleted) and with an intravenous injection of 1-2x10^6^ IL-2 activated syngeneic NK cells subsequently after successful surgery (NK cell injected). An additional group of recipients was treated with a daily injection of CsA (1.25 mg/kg body weight). (B) Infiltration of allogeneic grafts of Lew wt recipients without further treatment and after NK cell depletion or NK cell injection after graft rejection. The grafts were predominantly invaded by monocytes/macrophages, which were only marginally affected by NK cell manipulation. Of note, NK cell depletion caused a decrease of NKR-P1A^+^ (NK cells and NKT cells) cells below the baseline of syngeneic controls whereas the NKR-P1A/B^+^ (NK cells, NKT cells and Monocytes) cells were unaffected by the depletion (grey arrows), indicating abundant infiltration of NKR-P1B^+^ monocytes. (C) Kaplan-Meier presentation of allogeneic graft survival in Lew.1a rats treated accordingly to Lew wt recipients in (A). Contrary to the fast rejection model, Lew.1a recipients revealed accelerated rejection in the absence of NK cells and a reverse effect after injection of syngeneic NK cells, which induced graft survival over the complete observation period indicating establishment of graft acceptance. (D) Allogeneic graft infiltration of CD4^+^, CD68^+^, TCR^+^ and NKR-P1A/B^+^ expressing cells in Lew.1a recipients was only slightly elevated when compared to syngeneic grafts. However, interestingly and in contrast to Lew wt recipients the NK cell-depleted Lew.1a rats revealed a diminished fraction of NKR-P1A/B^+^ cells, indicating that in these recipients NK and NKT cells were the dominant population among these positive cells (grey arrow). Results are presented as mean ± SEM of at least 5 individuals per group. Significance is indicated with * for p-values ≤ 0.05, ** for p-values ≤ 0.005 and *** for p-values ≤ 0.0005. If no asterisk is shown in the respective graph the results were not significantly different.

Fig 4A shows the outcome of this respective treatment in Lew wt rats engrafted with Lew.1a hearts. Neither depletion nor injection of syngeneic NK cells could influence the course of rejection. Of note, daily administration of subtherapeutic doses of CsA (1.25 mg/kg body weight) led to a decline of CD4^+^ and CD8^+^ T cells and significantly prolonged the rejection of the grafts, indicating that the rejection is based on T cell activity (S4 Fig). The graft infiltrating cells were mainly T cells, macrophages and monocytes, whereas the latter dominated the infiltration of the allogeneic grafts (Fig 4B, S5A Fig).

The evaluation of the histological sections was based on the respective infiltration of syngeneic heart grafts (S5A and S5B Fig). The allografts in Lew wt recipients were invaded significantly above this baseline. The pattern of infiltration in untreated recipients was comprehensive and not restricted to certain local areas of the grafts. The same pattern could be observed in NK cell depleted and injected rats (data not shown). However, the treatment with CsA led to clusters of immune cells in the proximity of the ventricle and the edge of the muscle tissue (S5A Fig). Furthermore, we detected a significant increase of NK cell influx and a decrease of macrophages in the composition of infiltrating cells, whereas T cells revealed a slight increase compared to untreated rats (S5B and S5C Fig). Treatment of Lew wt recipients with NK cell depleting antibodies had only marginal effects on the graft infiltration, except for a significant decrease of NK cells among the infiltrating cells (Fig 4B). Injection of NK cells on the other hand had a surprising effect: While NKR-P1A/B^+^ (monocytes/NK and NKT cells) cells increased in the heart tissue, influx of NKR-P1A (NK and NKT cells) single positive cells was significantly reduced, when compared to syngeneic controls, which reflects a broad increase of monocytes into the graft tissue in the presence of additional syngeneic NK cells (Fig 4B).

In general, broad graft infiltration was already observed three days post HTx, especially with a significant influx of CD68^+^ macrophages and NKR-P1A/B^+^ monocytes when compared to the time of graft rejection (S5D Fig).

In contrast to the observations made in Lew wt recipients, the manipulation of NK cells revealed a key function of these cells in the regulation of rejection of Lew.1u7B heart grafts by Lew.1a recipients (Fig 4C). Depletion of recipient NK cells significantly reduced allograft survival to a mean survival of 15 days, whereas injection of syngeneic NK cells completely abrogated allograft rejection. Of note, the recipients received no other treatment for immunosuppression or foregoing immunization; solely the absence or enforced presence of NK cells induced the accelerated rejection and long-term graft survival, respectively. Histological analyses of the grafts revealed increased infiltration of syngeneic grafts when compared to syngeneic grafts of Lew wt recipients. Although an increase of cell influx in allogeneic grafts of untreated Lew.1a recipients was evident at the time of rejection, we observed a significant decrease of immune cells upon NK cell depletion, especially of the NKR-P1A/B^+^ cell population, despite an accelerated rejection (S5E Fig).

However, since additional infiltration of allogeneic grafts was only slightly above the baseline of syngeneic grafts, analysis by immunohistochemistry was insufficient in providing detailed changes depending on the performed intervention. We therefore decided to prepare heart tissue for flow-cytometry for further analysis of distinct lymphocyte subsets, despite the risk of selectively losing specific cell populations during tissue preparation.

### The injection of NK cells in Lew.1a recipients reduced the number of infiltrating monocytes and activated T cells drastically

**Fig 5:**
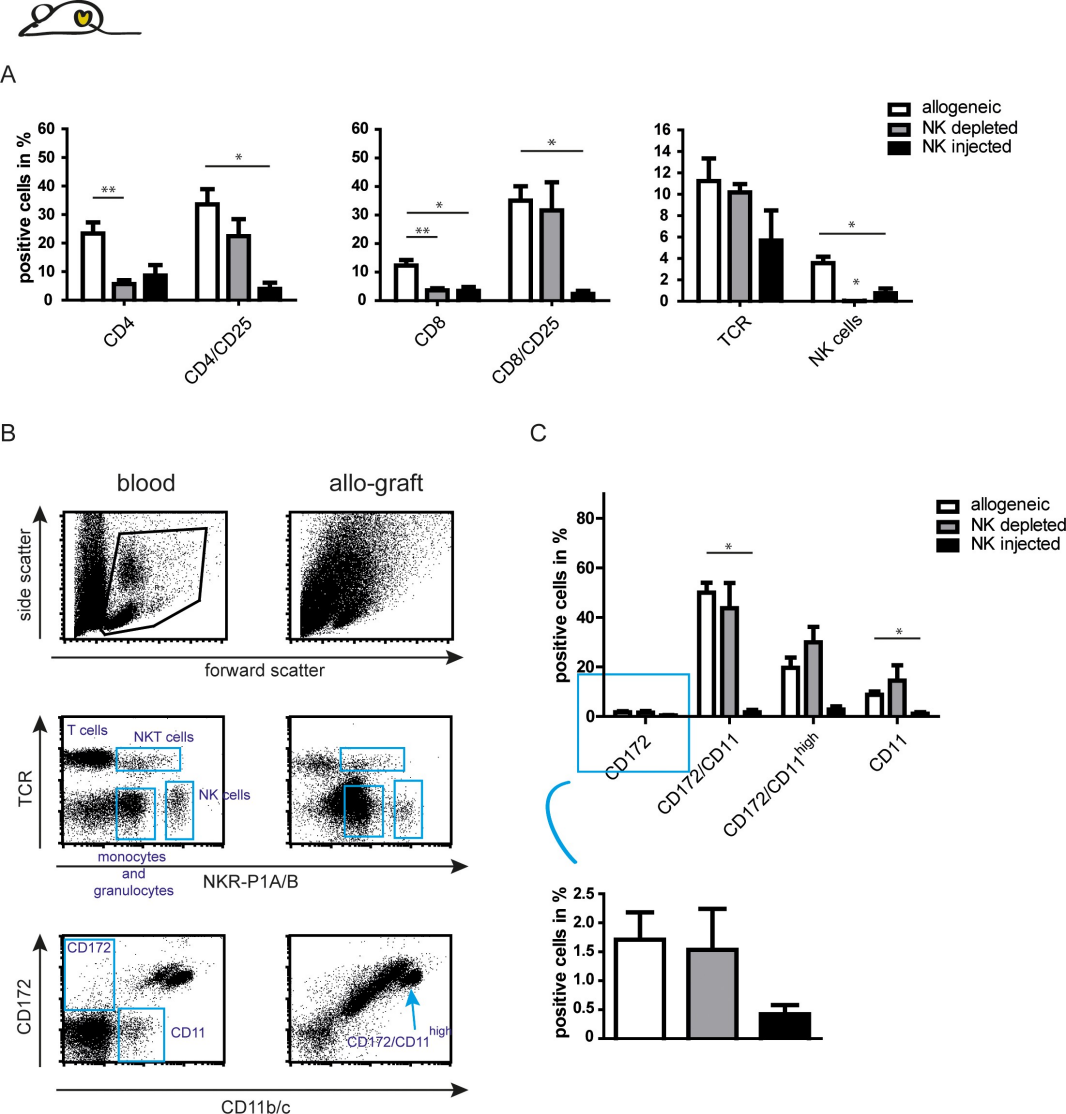
Graft infiltrating cells in Lew.1a recipients are affected by injection of activated syngeneic NK cells. (A) Flow cytometric analysis of Lew.1a derived infiltrates in allogeneic Lew.1u7B grafts in the specified group. In grafts of untreated Lew.1a recipients we observed increased fractions of CD4^+^, CD8^+^ T cells and especially of activated T cells (CD25^+^). These percentages did not correlate with the analysis of TCR^+^ cells, however, activated T cells often internalise their TCR, which might be an explanation for the discrepancy [26]. NK cells invaded the graft tissue only moderately. Depletion of NK cells led to a significant reduction of CD4^+^/ CD25^-^ and CD8^+^/CD25^-^ cells, whereas the influx of activated T cells was unaffected. The injection of syngeneic NK cells on the other hand, almost completely abrogated the infiltration of activated T cells into the graft. (B) Dot blots of lymphocytes and granulocytes in the blood and allografts defined by TCR vs. NKR-P1A/B and CD172 vs. CD11b/c expression of untreated recipients. Monocytes were among the major graft infiltrating cells and could be further discriminated according to the MFI of the CD11b/c expression. (C) Summary of the flow cytometric analysis of CD172/CD11b/c expressing cells in the respective group. Graft rejection, in particular after NK cell depletion, is accompanied by increased percentages of CD172^+^/CD11b/c^high^ cells. Injection of syngeneic NK cells impeded the invasion of macrophages/monocytes into the heart tissue. Results are presented as exemplary dot blots or as mean ± SEM of at least 5 individuals per group. Significance is indicated with * for p-values ≤ 0.05, ** for p-values ≤ 0.005 and *** for p-values ≤ 0.0005. If no asterisk is shown in the respective graph the results were not significantly different.

Flow cytometric analysis of heart infiltrating cells in Lew.1a rats exposed that the majority of infiltrating T cells were CD25^+^. The depletion of recipient NK cells did not significantly alter the population of CD4/CD25^+^ and CD8/25^+^ T cells despite an accelerated rejection. A substantial reduction of these cells however was observed after injection of NK cells, which diminished all infiltrating cell populations in comparison to untreated recipients (Fig 5A). Of note, the decrease of CD25^+^ T cells could not be solely attributed to either activated T cells or T_reg_ since flow cytometric analysis of FoxP3 expression revealed a distribution of T_reg_ among all CD25 expressing T cells in both recipient strains (S6 Fig). Therefore, the decrease of CD25^+^ T cells could account for both populations.

A reduced graft infiltration was also observed for the most abundantly detected cell population in the heart tissue: CD172^+^/CD11b/c^+^ monocytes and macrophages. Of note, a comparison of dot blots of blood circulating and heart infiltrating immune cells revealed that all populations detected in the blood were also present in the graft (Fig 5B). The CD172^+^/CD11b/c^+^ cells could be divided into two populations with respect to their predominant localization in blood and grafts. While the majority of CD172^+^/CD11b/c^+^ cells in the blood displayed a phenotype with a high mean fluorescence intensity of CD11 (termed here CD172^+^/CD11b/c^high^), there was a considerable CD172^+^/CD11b/c^+^ population among the graft infiltrating cells with a lower mean fluorescent intensity (MFI) of CD11, when compared to the fraction of CD172^+^/CD11b/c^high^ cells (termed here CD172^+^/CD11b/c^low^). Interestingly, there was a correlation of accelerated rejection and detected cell populations: NK cell depletion caused increasing percentages of CD172^+^/CD11b/c^high^ cells with simultaneous decrease of CD172^+^/CD11b/c^low^ cells. As assumed for the rejection process in Lew wt recipients, cells of the monocyte/macrophage fraction seemed to be critically associated with the rejection of the allogeneic graft in Lew.1a recipients. This is also reflected by the observations made after injection of syngeneic NK cells in Lew.1a recipients: all CD172 and CD11b/c positive cells (CD11b/c^low^ and ^high^) were considerably reduced (Fig 5C).

## Discussion

Although more commonly known for their pivotal role in cytotoxic and inflammatory processes, NK cells can also exhibit a strong regulative capacity towards T cells. This characteristic feature is not restricted to certain infections or immunological triggers. For instance, in mice it was reported for models of viral infections [27,28] and after allogeneic skin transplantation [29,30]. Previously, our workgroup has demonstrated the regulatory effects of rat NK cells on the onset of naïve T cells [16]. To evaluate the immunological significance of our *in vitro* results, we performed transplantation of allogeneic heart grafts in two strain combinations, characterized by a fast and a more prolonged course of rejection, respectively [25].

Despite providing further evidence for the distinct characteristic of NK cells to regulate T cell responses *in vitro*, we were surprised to learn that the manipulation of recipient NK cells either by depletion or injection, did not influence the course of fast rejection *in vivo*. This is most probably due to the severe activation of the adaptive and innate immune system upon allogeneic engraftment.

It is worth noting that, the fast and severe infiltration of Lew.1a grafts in Lew wt recipients by macrophages and monocytes (as precursors of tissue macrophages) raised doubts, that the rejection in this specific strain combination is predominately T cell mediated. However, the depletion of monocytes/macrophages by administration of Clodronate did not influence rejection (data not shown), whereas subtherapeutic administration of CsA significantly prolonged the course of rejection.

In conclusion, NK cells did not exhibit a central role in this rigid experimental setting of fast rejection of allogeneic heart grafts. Accordingly, previous studies have implicated that allogeneic grafts, provoking a strong immune response, are rejected independently of the presence of NK cells [4]. In contrast, tolerance mediated by NK cells was shown to be induced in a moderate course of rejection, if T cell activity is hindered by co-stimulatory blockage [7]. In line with these reports, the presence or absence of NK cells in the second strain combination, characterized by a more prolonged rejection, significantly influenced the survival of the graft: Depletion of recipient NK cells led to a significantly accelerated loss of graft function, whereas injection of syngeneic NK cells completely prevented graft rejection, without simultaneous immunosuppression.

Of note, the applied strain combination showed occasional indefinite graft survival for untreated recipients, indicating an exceptional immunological setting. Specific strain combinations with similar observations of prolonged graft survival, despite fully disparate MHC, were identified in the past [31]. However the underlying mechanisms explaining these results were not further clarified.

In past experiments immunization of Lew.1a recipients with Lew.1u7B derived blood [32] or skin [33] led to complete tolerance of Lew.1u7B heart grafts without further treatment, which was demonstrated to be infectious for naïve recipients. This is especially noteworthy, since others postulated that donor-specific transfusion (DST)-mediated tolerance can only be induced by depletion of recipient CD4^+^ T cells [34] or mild gamma-irradiation of recipients [35]. As an explanation for these observations, the authors concluded that by attenuating the initial trigger for an allogeneic immune response, the induction of a repertoire of CD4^+^ regulatory T-cells is facilitated, which results in a highly efficient mechanism to maintain tolerance, even after several “infectious transfers”. Interestingly, TGF-β seems to be crucially involved in this procedure [33], which would be in line with our previous data, revealing that suppression of T cells by NK cells is mediated directly by secretion of TGF-β [16]. Therefore, we assessed *ex vivo* mRNA expression of selected cytokines in draining LN cells from Lew.1a and Lew wt rats after exposure to donor (Lew.1u7B and Lew.1a respectively) derived allogeneic heart cells. We found similar TGF-β expression in both strain combinations, but a more inflammatory cytokine profile in Lew.1a recipients with enhanced expression of IFN-γ, whereas Lew wt recipients tended to an expression of a more suppressive profile, with expression of IL-10 and FoxP3. A possible explanation could be, that a strong immune response provokes a higher induction of suppressive cytokines in general, since mitigating a highly inflammatory immune response is of high priority for the host, as has been demonstrated by Lee et al. in mouse model of CMV [36].

While it is a common notion, that regulatory T cells play a central role in inducing and maintaining graft acceptance, our flow cytometric analyses to define the role of regulatory T cell populations (CD4^+^CD25^+^FoxP3 as defined by Nessi et al. in rats [37]) in selected experiments, did not reveal significant alterations of this cell population, leading to the assumption that these are not major contributors to the process of graft acceptance in our setting (preliminary data not shown). Accordingly, Lair et al. reported on tolerance transfer in the same transplantation model and rat strain combination as used in our experiments (Lew.1w ≙ Lew.1u → Lew.1a), providing proof for blood non-T cells as effective mediators for induction of indefinite graft survival upon adoptive transfer, as opposed to a transfer of blood T cells, which failed to induce tolerance in this special setting [38].

As stated above, monocytes and macrophages were the predominant graft infiltrating cell population and were detected as early as 3 days post HTx, which is in line with previous reports [39,40,41]. The simultaneous expression of CD4 and CD68 points towards a CD4^+^ population of macrophages that invades the grafts, as was reported by Wallgren et al. in the past [41]. The infiltrating monocytes and macrophages were shown to express inflammatory molecules, like inducible NO synthase (iNOS) and TNF-α, which can damage the function of the graft, eventually resulting in rejection [40]. The participation of graft infiltrating macrophages/monocytes could also be tracked in the Lew.1a recipients engrafted with hearts of Lew.1u7B, whereby the presence of CD172^+^/CD11b/c^+^ cells was abundant in untreated recipients rejecting the grafts. For dendritic cells (DC) it has been shown that the CD172^+^/CD11b^high^ expressing cells enhance inflammation in LN and directly at the site of inflammation [42]. Upon depletion of NK cells and concomitant accelerated course of rejection, we observed a significant increase of CD172^+^/CD11b/c^high^ expressing cells in the graft, whereas injection of NK cells abrogated the occurrence of these cells. We assume that these observations are caused by an attenuated activation of T cells mediated by NK cells, leading to a reduction of signals attracting inflammatory cells into the graft tissue. This is supported by the almost complete disappearance of CD25^+^ (activated) T cells in the heart grafts after injection of syngeneic NK cells.

Furthermore, we believe that the attenuation of T cell activation occurs in the draining LN, as has also been demonstrated by Laffont et al. in a model of mouse skin transplantation [30]. This is indicated by the significant increase of NK cells in draining LN of Lew.1a, but not of Lew wt rats upon subcutaneous placement of allogeneic heart cells into the pinna. Lew.1a rats reacted with a significant increase of NK cells in draining LN in comparison to naïve rats and suppressed the T cell response upon allogen-specific re-stimulation significantly, which could not be observed in Lew wt rats. We assume that this attenuated response in combination with the pronounced characteristic of NK cell homing to draining LN in Lew.1a is the key of tolerance induction in these rats. This is additionally suggested by the significant decrease of circulating NK cells in untreated Lew.1a recipients, rejecting the allogeneic heart grafts.

The mechanism of this suppressed T cell activity can only be assumed, since cytokine profiles did not give clear indications of an immunosuppressive environment. Aside from suppressive cytokine production, direct lysis of activated T cells (possibly via perforin-dependent mechanisms) could be hypothesized, as was reported by several authors [29,30], however previous analyses for NK cell cytotoxicity in the two implemented strains in our laboratory did not reveal significant differences between the two strains and flow cytometric analysis for CD107a expression in experiments did not indicate NK-cell mediated lysis of target cells (data not shown).

In this study, we provide evidence that NK cells are regulative agents, able to control T cell activity during rejection of completely MHC-disparate grafts. This observation however, was dependent on the chosen strain combination. The diversity of animal models used to evaluate the role of NK cells in graft rejection might provide a reasonable explanation, why such diverse and to some extent contrary outcomes are postulated, ranging from no participation [3,4,5], to participation which is outweighed by T cells [8,43] and finally with active participation [7,10,13]. The direct comparison of the two presented strain combinations in this study gives an indication that the degree of allogeneic immune activation is crucial, when evaluating participation of specific cell populations in the course of rejection and leads us to following conclusion: NK cells participate and are able to influence the course of solid organ rejection, as long as the stimulation of T cells is of moderate nature.

## Supporting information

S1 Fig Results from flow cytometric analysis in Lew wt and Lew.1a recipients. (A) Percentages of NK cells were assessed in cervical (cerv) and mesenterial (mes) LN, the spleen and blood by flow cytometry in rats divided in rejecting and non-rejecting recipients as it has been already shown in Fig 1C with an additional comparison to naïve rats, revealing a tendency towards reduced NK cell fractions in rejecting recipients when compared to naïve rats. (B) Flow cytometric analysis of tissue samples derived from Lew wt and Lew.1a recipients showing the relative frequencies of T cells among lymphocytes derived from cervical and mesenteric LN (white and black bars) and also from spleen and blood (bright and dark grey bars) of the respective group. In recipients of both strains a decrease of T cells frequencies after allogeneic engraftment was observed in the majority of the analyzed samples. In Lew wt recipients this decline was further accentuated by injection of syngeneic NK cells, whereas NK cell depletion led to a significant incline of T cells in the blood and in the spleen.
(TIF)

S2 Fig Proliferation of draining lymph node cells upon subcutaneous placement of allogeneic heart muscle cells. The dot blots show the CFSE-based proliferation of cervical (draining) lymph node cells of Lew wt and Lew.1a rats after subcutaneous placement of allogeneic heart muscle cells (derived from Lew.1a and Lew.1u7B, respectively) and 6 days after *in vitro*-stimulation with splenocytes from the respective donor strain (specific re-stimulation) or with splenocytes from a third party strain (for Lew wt responder → Lew.1u7B cells and for Lew1.a responder → Lew wt cells; unspecific re-stimulation).
Whereas Lew wt T cells responded with an enhanced proliferation to the specific re-stimulation in comparison to an unspecific stimulus, T cells derived from Lew.1a rats showed a significant reduced proliferation upon specific re-stimulation. This reduction of proliferation was mainly based on a reduced number of activated T cells in general and not by means of cell divison as indicated by the dot blots.
Of note, the ability to regulate the T cell responses by NK cells in Lew.1a rats is promoted by a weak induction of proliferation through Lew.1u7B cells in Lew rat strains in general, and especially in Lew.1a rats.
(TIF)

S3 Fig Relative frequencies of NK cells in Lew wt and Lew.1a recipients upon intervention. Flow cytometric analysis of the relative frequencies of NK cells among lymphocytes derived from cervical and mesenteric LN (white and black bars) and from spleen and blood (bright and dark grey bars) of the respective group in Lew wt and Lew.1a recipients, after rejection and at the end of the observation period, respectively. While NK cell depletion was successful in both strains and in all analyzed compartments, untreated Lew wt recipients, unlike Lew.1a recipients, showed significantly increased NK cell ratios upon engraftment in spleen and blood.
(TIF)

S4 Fig T cell and NK cell distribution in Lew wt recipients upon syngeneic and allogeneic engraftment. Flow cytometric analysis of relative percentages of CD4^+^ cells, CD8^+^ cells and NK cells of Lew wt recipients upon syngeneic and allogeneic engraftment with and without subtherapeutic CsA treatment. Recipients receiving CsA treatment showed significantly elevated NK cell ratios particularly in the lymphatic tissue, whereas CD4^+^ and CD8^+^ T cells showed significant decline, especially in the blood.
(TIF)

S5 Fig Graft invasion by immune cells of Lew wt recipients. (A) Infiltration of syngeneic Lew wt and allogeneic Lew.1a heart grafts of untreated and CsA treated recipients, respectively, by CD4^+^, CD68^+^ (macrophages), TCR^+^ and NKR-P1A/B^+^ cells. (B) Cell staining via mAb HT30 revealed that NK (and NKT) cells were rarely detected in syngeneic and also in allogeneic grafts suggesting a minor role of these cells in local immune responses in the graft (arrows). However, upon subtherapeutic CsA treatment grafts revealed increased NK (and NKT) cell fractions. (C) Infiltration scores of allogeneic grafts of untreated Lew wt recipients and after CsA treatment based on syngeneic graft infiltration. (D) Infiltration scores 3 days post transplantation of allogeneic grafts derived from untreated and NK cell depleted recipients. The bright grey bars indicate the infiltration scores after rejection corresponding to Fig 4B. The graph highlights the early graft invasion by macrophages in untreated recipients, especially when compared to recipients after NK cell depletion. (E) Infiltration of syngeneic and allogeneic grafts of Lew.1a recipients assessed by nuclear hematoxylin staining. Due to the intense infiltration of syngeneic grafts it was difficult to analyse additional infiltration of allogeneic grafts. Of note, the degree of infiltration in untreated rejecting and non-rejecting Lew.1a recipients (see Fig 1C and 1D) revealed no significant differences, which is why the allogeneic group was presented undivided as one group.
(TIF)

S6 Fig Distribution of CD25^+^/FoxP3^+^ T_reg_ in draining lymph nodes of Lew wt and Lew.1a rats. (A) Flow cytometric analyses were performed using cervical lymph node cells of naïve Lew wt and Lew.1a rats with a distinct gate on the lymphocyte population (including lymphoblasts). Detailed assessment of CD25^+^CD4^+^ T cells revealed a homogenous distribution of FoxP3^+^ cells. Therefore, a consistent discrimination between activated T cells and (FoxP3 expressing) T_reg_ cannot be made, since additional FoxP3 staining was not performed for all samples. Naturally occurring T_reg_ cells in rat tend to express higher levels of CD25, than activated T effector cells, however this is not always a valid tool for discrimination of these cell populations as was pointed out in both recipient strains.
(TIF)

S1 File ARRIVE guidelines. (DOCX)
