# Supplementary figures and images for: Recipient natural killer cells alter the course of rejection of allogeneic heart grafts in rats
Source: PLoS One. 2019 Aug 22;14(8):e0220546. doi: 10.1371/journal.pone.0220546 (PMC6705777; doi:10.1371/journal.pone.0220546)

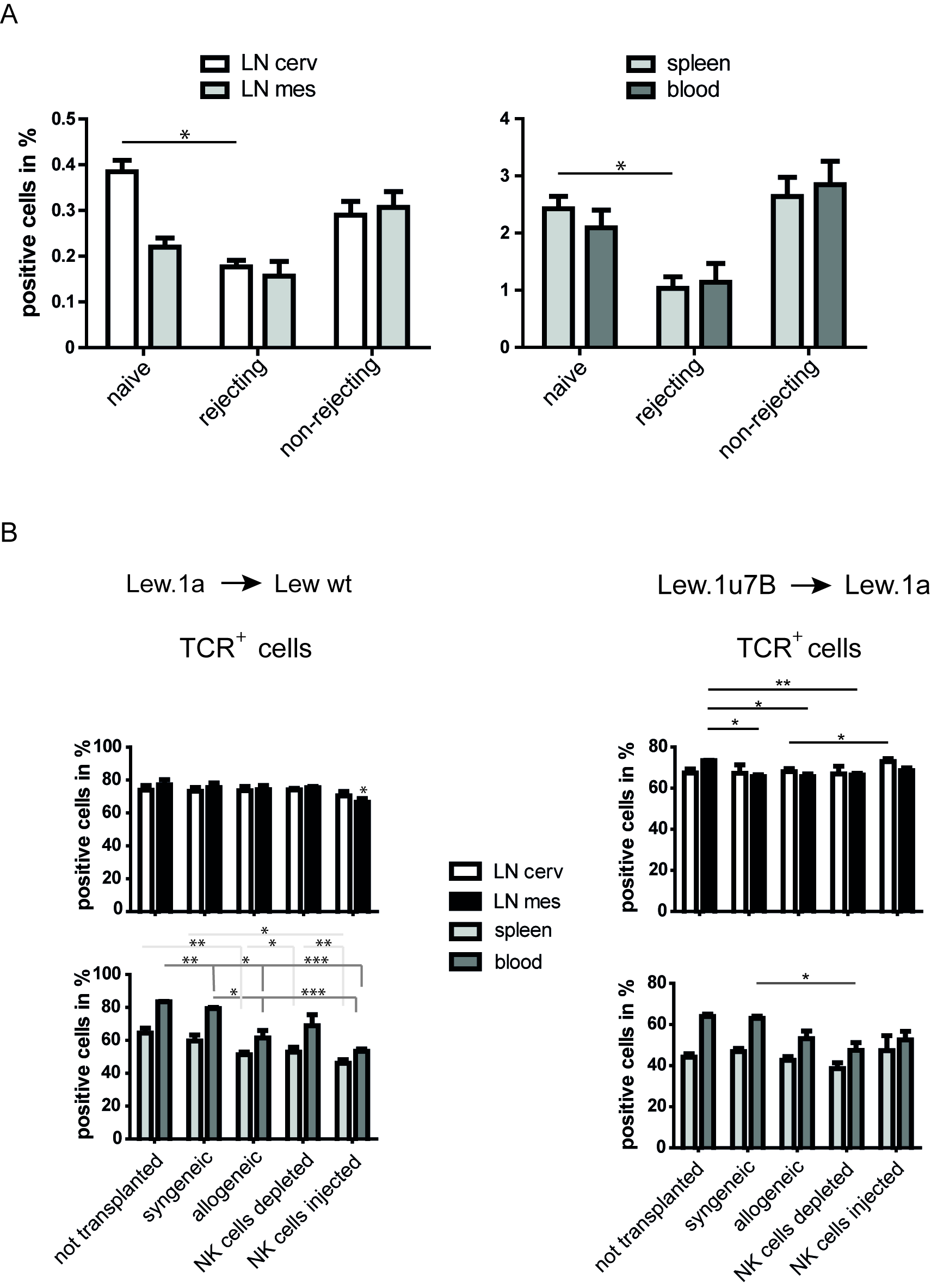

Supplement: S1 Fig — (A) Percentages of NK cells were assessed in cervical (cerv) and mesenterial (mes) LN, the spleen and blood by flow cytometry in rats divided in rejecting and non-rejecting recipients as it has been already shown in Fig 1C with an additional comparison to naïve rats, revealing a tendency towards reduced NK cell fractions in rejecting recipients when compared to naïve rats. (B) Flow cytometric analysis of tissue samples derived from Lew wt and Lew.1a recipients showing the relative frequencies of T cells among lymphocytes derived from cervical and mesenteric LN (white and black bars) and also from spleen and blood (bright and dark grey bars) of the respective group. In recipients of both strains a decrease of T cells frequencies after allogeneic engraftment was observed in the majority of the analyzed samples. In Lew wt recipients this decline was further accentuated by injection of syngeneic NK cells, whereas NK cell depletion led to a significant incline of T cells in the blood and in the spleen. (TIF) [file pone.0220546.s001.tif]

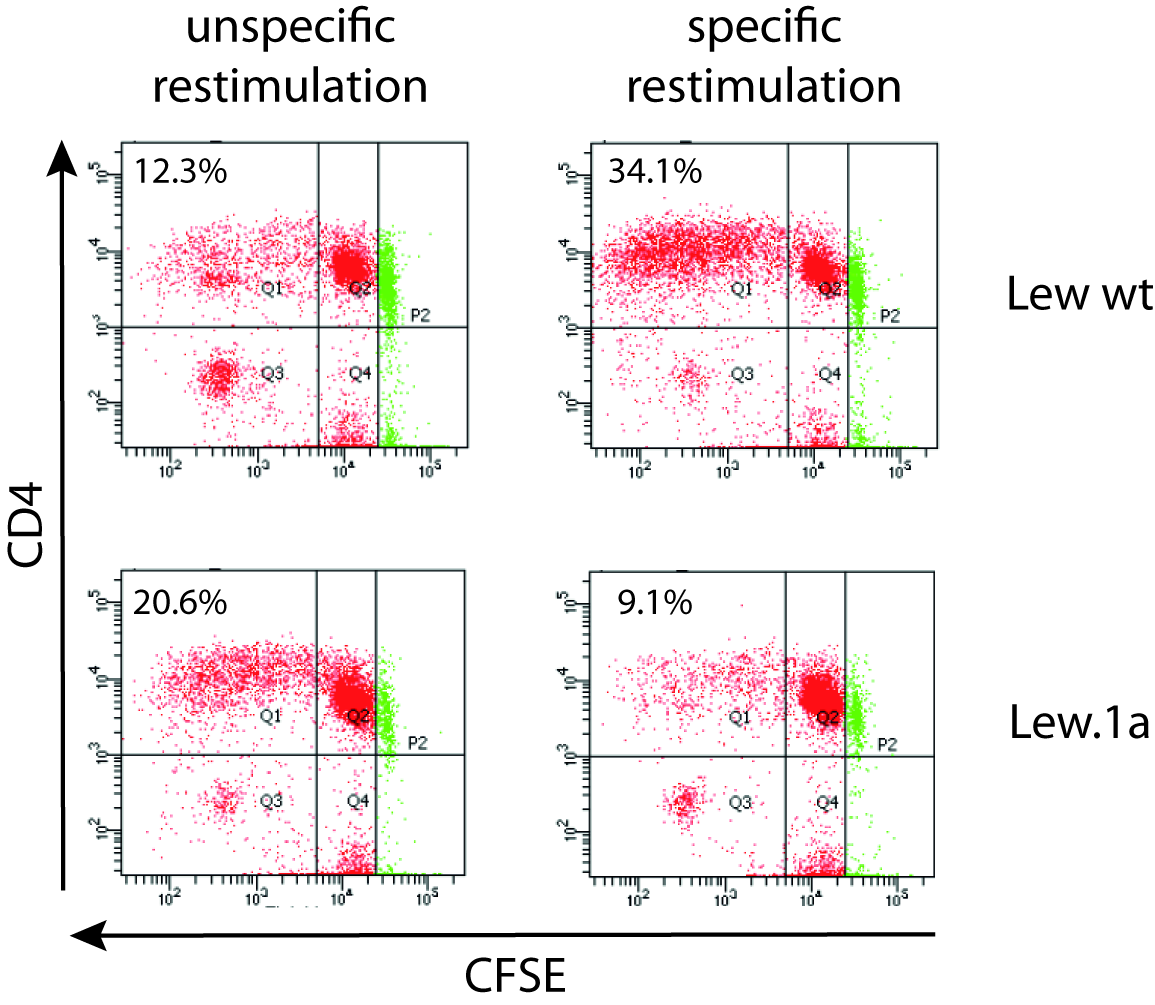

Supplement: S2 Fig — The dot blots show the CFSE-based proliferation of cervical (draining) lymph node cells of Lew wt and Lew.1a rats after subcutaneous placement of allogeneic heart muscle cells (derived from Lew.1a and Lew.1u7B, respectively) and 6 days after in vitro-stimulation with splenocytes from the respective donor strain (specific re-stimulation) or with splenocytes from a third party strain (for Lew wt responder → Lew.1u7B cells and for Lew1.a responder → Lew wt cells; unspecific re-stimulation). Whereas Lew wt T cells responded with an enhanced proliferation to the specific re-stimulation in comparison to an unspecific stimulus, T cells derived from Lew.1a rats showed a significant reduced proliferation upon specific re-stimulation. This reduction of proliferation was mainly based on a reduced number of activated T cells in general and not by means of cell divison as indicated by the dot blots. Of note, the ability to regulate the T cell responses by NK cells in Lew.1a rats is promoted by a weak induction of proliferation through Lew.1u7B cells in Lew rat strains in general, and especially in Lew.1a rats. (TIF) [file pone.0220546.s002.tif]

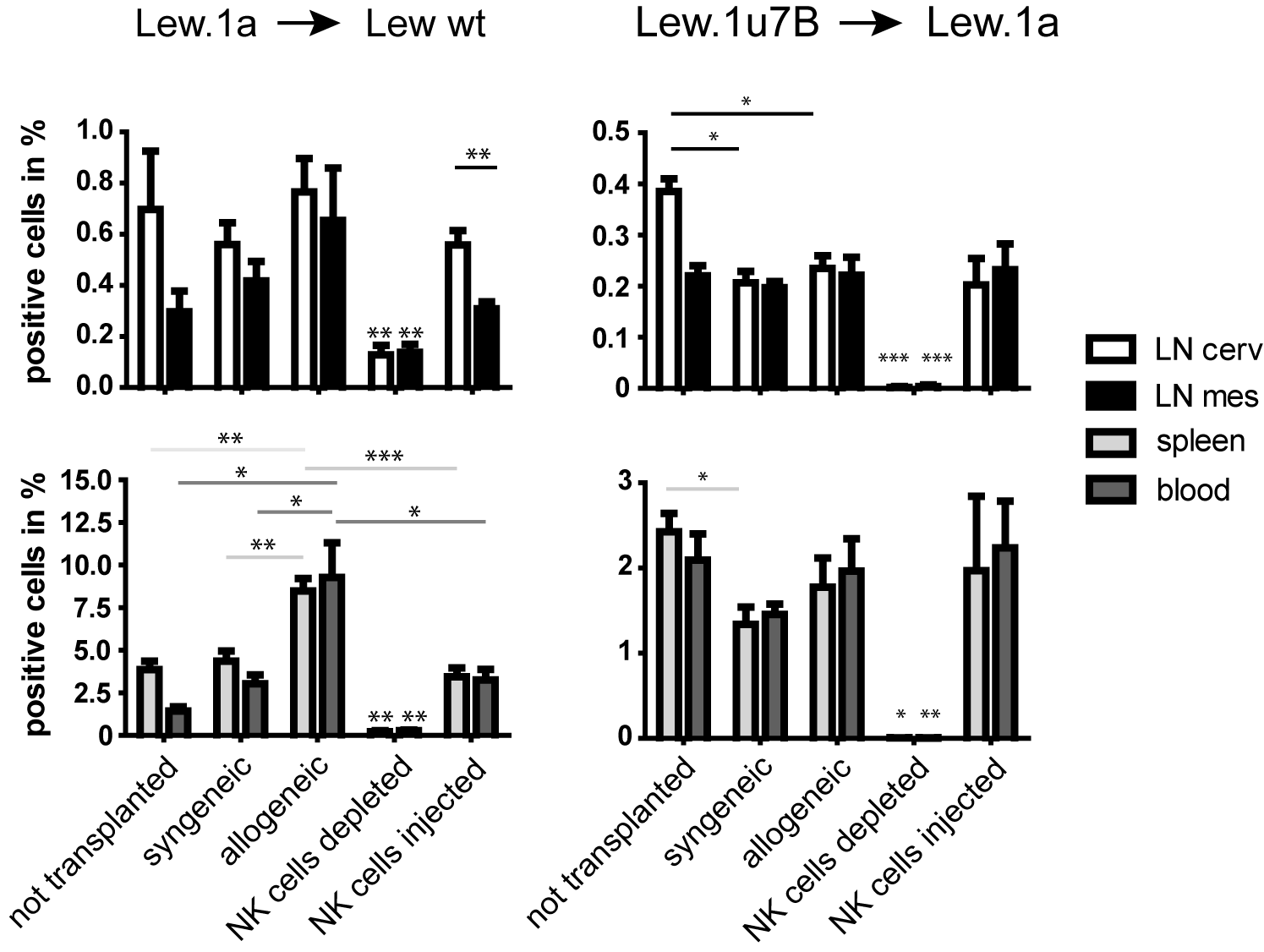

Supplement: S3 Fig — Flow cytometric analysis of the relative frequencies of NK cells among lymphocytes derived from cervical and mesenteric LN (white and black bars) and from spleen and blood (bright and dark grey bars) of the respective group in Lew wt and Lew.1a recipients, after rejection and at the end of the observation period, respectively. While NK cell depletion was successful in both strains and in all analyzed compartments, untreated Lew wt recipients, unlike Lew.1a recipients, showed significantly increased NK cell ratios upon engraftment in spleen and blood. (TIF) [file pone.0220546.s003.tif]

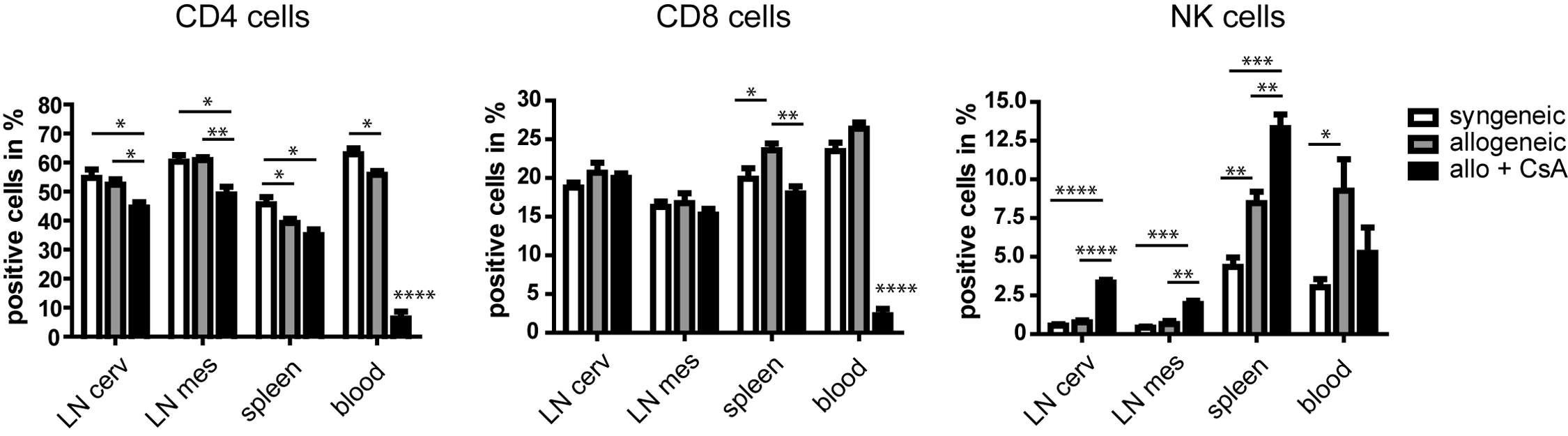

Supplement: S4 Fig — Flow cytometric analysis of relative percentages of CD4+ cells, CD8+ cells and NK cells of Lew wt recipients upon syngeneic and allogeneic engraftment with and without subtherapeutic CsA treatment. Recipients receiving CsA treatment showed significantly elevated NK cell ratios particularly in the lymphatic tissue, whereas CD4+ and CD8+ T cells showed significant decline, especially in the blood. (TIF) [file pone.0220546.s004.tif]

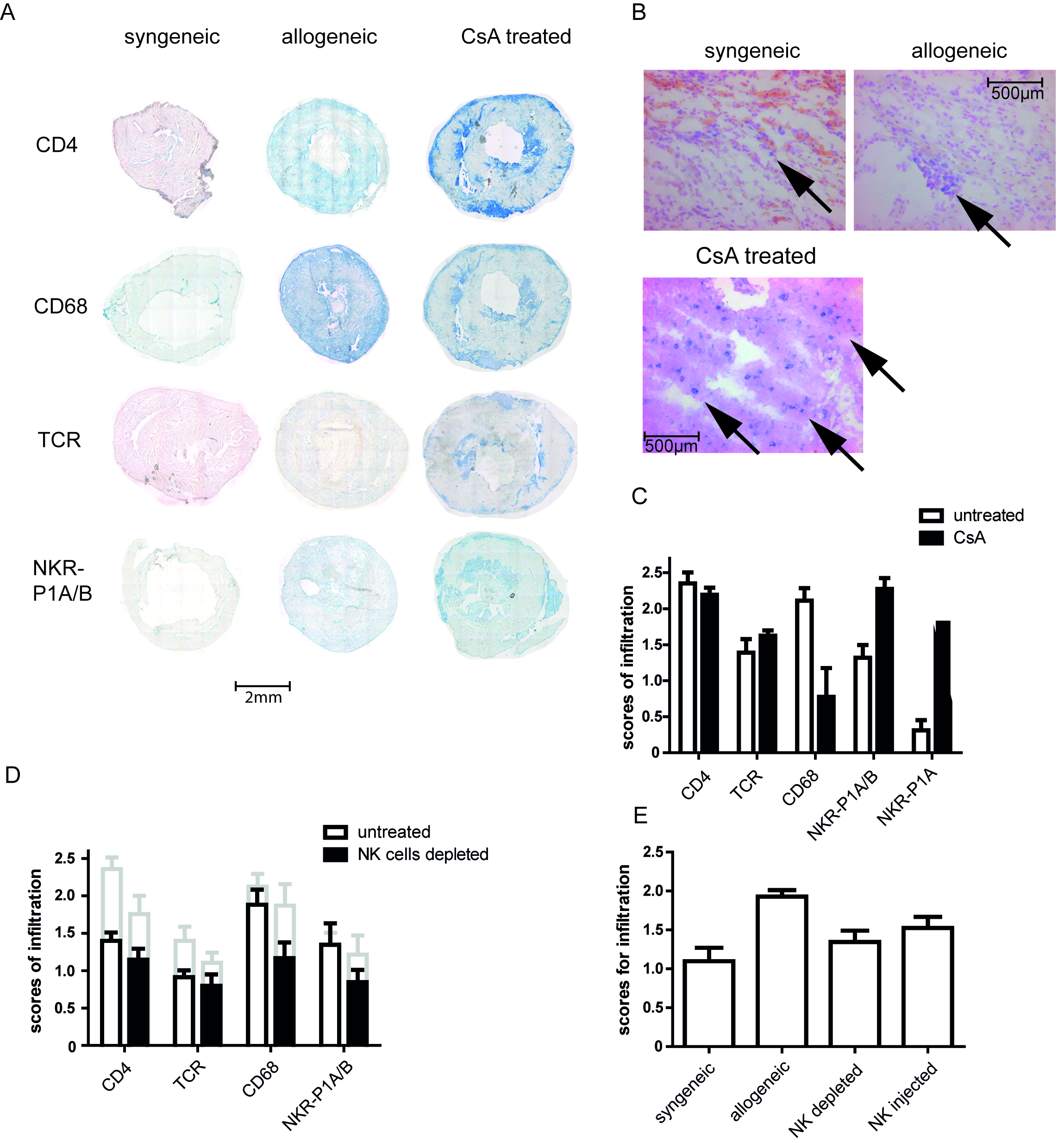

Supplement: S5 Fig — (A) Infiltration of syngeneic Lew wt and allogeneic Lew.1a heart grafts of untreated and CsA treated recipients, respectively, by CD4+, CD68+ (macrophages), TCR+ and NKR-P1A/B+ cells. (B) Cell staining via mAb HT30 revealed that NK (and NKT) cells were rarely detected in syngeneic and also in allogeneic grafts suggesting a minor role of these cells in local immune responses in the graft (arrows). However, upon subtherapeutic CsA treatment grafts revealed increased NK (and NKT) cell fractions. (C) Infiltration scores of allogeneic grafts of untreated Lew wt recipients and after CsA treatment based on syngeneic graft infiltration. (D) Infiltration scores 3 days post transplantation of allogeneic grafts derived from untreated and NK cell depleted recipients. The bright grey bars indicate the infiltration scores after rejection corresponding to Fig 4B. The graph highlights the early graft invasion by macrophages in untreated recipients, especially when compared to recipients after NK cell depletion. (E) Infiltration of syngeneic and allogeneic grafts of Lew.1a recipients assessed by nuclear hematoxylin staining. Due to the intense infiltration of syngeneic grafts it was difficult to analyse additional infiltration of allogeneic grafts. Of note, the degree of infiltration in untreated rejecting and non-rejecting Lew.1a recipients (see Fig 1C and 1D) revealed no significant differences, which is why the allogeneic group was presented undivided as one group. (TIF) [file pone.0220546.s005.tif]

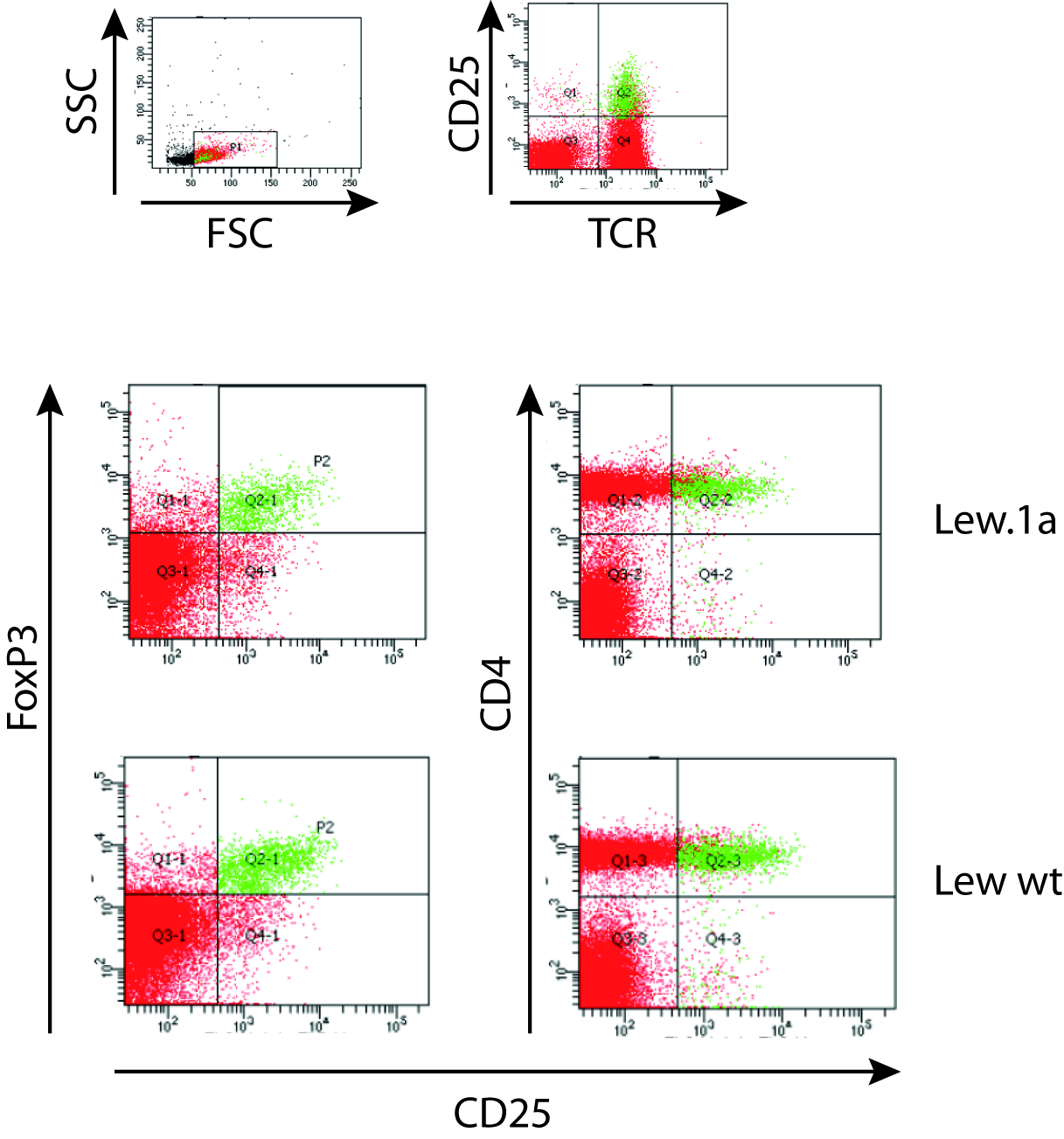

Supplement: S6 Fig — (A) Flow cytometric analyses were performed using cervical lymph node cells of naïve Lew wt and Lew.1a rats with a distinct gate on the lymphocyte population (including lymphoblasts). Detailed assessment of CD25+CD4+ T cells revealed a homogenous distribution of FoxP3+ cells. Therefore, a consistent discrimination between activated T cells and (FoxP3 expressing) Treg cannot be made, since additional FoxP3 staining was not performed for all samples. Naturally occurring Treg cells in rat tend to express higher levels of CD25, than activated T effector cells, however this is not always a valid tool for discrimination of these cell populations as was pointed out in both recipient strains. (TIF) [file pone.0220546.s006.tif]
