# Supplementary material for: Recipient natural killer cells alter the course of rejection of allogeneic heart grafts in rats
Source: PLoS One. 2019 Aug 22;14(8):e0220546. doi: 10.1371/journal.pone.0220546 (PMC6705777; doi:10.1371/journal.pone.0220546)
Supplement: S1 File — (DOCX) [file pone.0220546.s007.docx]

**ARRIVE Guideline Checklist**

regarding the study:

***“Recipient Natural killer cells alter the course of rejection of allogeneic heart grafts in rats”***

by Beetz O. and Kolb J. et al. (PONE-D-19-01634R1)

Item 1-4 Are included in the ***Title, Abstract and Introduction***

**Methods**

Item 5 Section “Material and Methods”, Paragraph “Ethics”

Item 6 Section “Material and Methods”, Paragraph “Study design”

Item 7 Section “Material and Methods”, Paragraphs “Heterotopic Heart Transplantation”, “Treatment of rats prior and after transplantation”, “Subcutaneous placement of heart cells in the ear”

Item 8 Section “Material and Methods”, Paragraph “Animals”

Item 9 Section “Material and Methods”, Paragraph “Animals”

Item 10 Section “Material and Methods”, Paragraph “Study design” and in each Legend to the respective figure.

Item 11 Section “Material and Methods”, Paragraphs “Heterotopic Heart Transplantation”, “Treatment of rats prior and after transplantation”, “Subcutaneous placement of heart cells in the ear”

Item 12 Section “Material and Methods”, Paragraph “Heterotopic Heart Transplantation” and in each Legend to the respective figure.

Item 13 Section “Material and Methods”, Paragraph “Statistical analysis”

**Results**

Item 14-17 If applicable, these nformation are included in the text of our “Results” section

**Discussion**

Item 18-20 If applicable, these information are included in the text of our “Discussion” section.
